# Cimetidine: A Safe Treatment Option for Cutaneous Warts in Pediatric Heart Transplant Recipients

**DOI:** 10.3390/medsci6020030

**Published:** 2018-04-08

**Authors:** Bibhuti B Das, Kristin Anton, Nelia Soares, Susan Riojas, Jodi Mcdermott, Leah Knox, Susan Daneman, Bao N Puente

**Affiliations:** 1 Joe DiMaggio Children’s Hospital Heart Institute, Memorial Health Care, Hollywood, FL 33021, USA; 2 Children’s Health Heart Institute, Dallas, Texas 75235, USA; Kristin.Anton@Childrens.com (K.A.); Nelia.Soares@childrens.com (N.S.); susan.riojas@childrens.com (S.R.); Jodi.Mcdermott@childrens.com (J.M.); Leah.Knox@childrens.com (L.K.); Susan.Daneman@childrens.com (S.D.); 3 Department of Pediatrics, UTSW Medical Center, Dallas, Texas 75235, USA; rbynmd@gmail.com

**Keywords:** cimetidine, cutaneous warts, pediatric heart transplant recipients

## Abstract

*Background and Objectives*: Immunosuppressed individuals are at particularly increased risk for human papilloma virus-related infections. The primary objective of our study is to determine if there are any adverse effects associated with high-dose cimetidine treatment. A secondary objective is to report our experience with cimetidine in the treatment of cutaneous warts in pediatric heart transplant recipients. *Methods and Results*: This was a retrospective observational study. A total of 8 pediatric heart transplant recipients diagnosed with multiple recalcitrant warts were the subject of the study. All patients were treated with cimetidine (30–40 mg/kg/day) in two divided doses for 3 to 6 month durations. All patients had complete resolution of their lesions except 1 patient who had no clinical improvement. Of these 8 patients, one had recurrence of warts at one year follow-up, which resolved with restarting cimetidine therapy. One patient who had only 3 months of cimetidine therapy had immediate relapse after cimetidine was stopped. None of them had significant change in their tacrolimus trough, serum creatinine, and alanine transaminase levels. No adverse events were reported except one patient experienced mild gynecomastia. *Conclusion*: Cimetidine can be a safe and alternative treatment option for multiple warts in pediatric heart transplant recipients.

## 1. Introduction

Cutaneous warts (verrucae) are caused by human papilloma virus (HPV), a double-stranded DNA papovavirus, which commonly affect children and adolescents and can be functionally and cosmetically disabling. Clinically, the four most common types of cutaneous warts are: the common wart (verruca vulgaris), plantar wart (verruca plantaris), flat wart (verruca plana), and genital wart (condyloma acuminatum). More than over 120 HPV types have been described. HPV 27, 57, 2, and 1 are the most prevalent HPV types in cutaneous warts in general population [1]. The prevalence of warts in the general population is unknown, the estimated peak incidence is 3–20%, occurring mostly between the ages of 9 and 16 years [2]. Presence of warts is directly proportional to duration of immunosuppression in solid transplant patients. In one case series, the prevalence for warts is 24.4% in 217 consecutive pediatric solid-organ transplant patients [3]. In another series of solid organ transplant patients, after 5 years of transplantation, 92% of patients were found to have warts and 65% have more than five warts each. DNA extraction from cutaneous warts showed that warts in solid organ transplant patients carried similar HPV types as the general population [4]. In immunocompetent patients, warts can often spontaneously regress. However, in patients with decreased cellular immunity (e.g., acquired immune deficiency syndrome or organ transplantation), warts become recalcitrant and persist due to immunosuppression [5]. 

No single agent has been universally efficacious in the treatment of warts. Treatment approaches to warts in immunocompromised hosts include duct tape, topical therapy with imiquimod, podophyllin, fluorouracil, cidofovir, intralesional and/or intramuscular interferon α-2b, surgical resection, and laser or cryotherapy but are often associated with recurrence and clinical failure [6]. Unfortunately, no therapeutic modality, alone or in combination, has been established as definitively superior to others. Therefore, several alternative treatment modalities for warts have been investigated. In particular, immunomodulatory agents targeted at the underlying HPV infection have produced the most encouraging outcomes. 

Cimetidine is a histamine-2 antagonist that is typically used for gastric acid suppression. The immunomodulatory effect of cimetidine at high dose (30–40 mg/kg/day) has been recognized and its clinical use as treatment for warts has been reported in small studies [7,8,9,10,11]. The results of these studies are inconsistent, with several open label studies reporting efficacy while others showed no advantage over placebo [12,13]. Cimetidine is well tolerated in all of these studies, without any major side effects. The safety of cimetidine treatment specifically in pediatric heart transplant recipients with cutaneous warts has not been reported in the literature. 

The primary objective of our study is to determine if any adverse effects are associated with high dose cimetidine treatment and the secondary objective is to report our experience with cimetidine in the treatment of cutaneous warts in pediatric heart transplant recipients. 

## 2. Methods

This was a retrospective chart review study of pediatric heart transplant recipients at Children’s Health, Dallas from 2012 to 2016 with the clinical diagnosis of cutaneous warts and treated with cimetidine. The study was approved by the Institutional Review Board by Children’s Medical Center Dallas and UTSW Medical Center (Dallas, Texas) on 22 March 2017, IRB #STU 0222015-029. Patients were selected according to the following inclusion criteria: (i) presence of clinically significant warts that were symptomatic including associated pain, (ii) discomfort or functional impairment, (iii) social stigma, or (iv) concern for cosmesis, for which patients had been subjected to one or more treatment approaches by destructive methods such as laser cauterization, duct tape, or application of podophyllin without resolution of clinical presentation. All patients were treated with cimetidine (30–40 mg/kg/day) in two divided doses for three to six months. Their electronic medical records were reviewed for demographic data, age at heart transplantation, immunosuppression regimen, type, and location of warts, and the disease course. We also reviewed the tacrolimus drug levels, renal (serum creatinine) and liver function (alanine transaminase) results pre- and post-cimetidine therapy. None of the patients had a history of an allergy to cimetidine.

## 3. Results

During the study period, a total of 8 patients with multiple cutaneous warts were included in this study. The demographic data including age and gender distribution, immunosuppression regimen, types of warts, duration of cimetidine therapy, and outcomes at both 6 months and at 1 year after completion of therapy were described in Table 1. All patients were diagnosed clinically and did not undergo specific HPV serotyping, except for patient #7 (Table 1). Duration of therapy was based on responsiveness to treatment. Seven patients completed 5 months of 30–40 mg/kg/day cimetidine therapy and all had complete resolution of their lesions except 1 patient who had no clinical improvement. The patient (patient #7, Table 1) had common warts in axillary area in addition to condyloma accuminata and polymerase chain reaction testing from the lesions were positive for HPV type 6 and 11. One patient (Patient #8, Table 1) who received only 3 months of cimetidine therapy had immediate relapse after it was stopped, which was successfully treated with additional 6 months of cimetidine therapy. 

Tacrolimus level pre- and post-cimetidine were 5 ± 4 ng/mL and 5 ± 6 ng/mL (*p* = 0.87) respectively. Likewise, serum creatinine (0.61 ± 0.23 mg/dL vs. 0.76 ± 0.27 mg/dL; *p* = 0.35) and liver enzyme alanine transaminase level (25.37 ± 7.6 vs. 23 ± 9.8 units/L; *p* = 0.67) did not change significantly pre- and post-cimetidine therapy. There were no reported adverse effects by the patients during the treatment course with cimetidine necessitating cessation of therapy. One patient (patient #3) had severe pruritus but she had also atopic dermatitis which was treated separately. One patient (patient #4) had mild gynecomastia, which resolved after 2 weeks of completion of his cimetidine therapy.

## 4. Discussion

Organ transplantation with immunosuppression therapy predisposes a patient to more extensive or recalcitrant cutaneous warts due to HPV infections [3]. Various reasons including lack of production of memory T cells to target HPV infection, failure of clonal expansion of lymphocytes to adequate stimulation, inability of T lymphocytes to traffic to sites of infection and weak effector response mechanism have been hypothesized [14]. In this study, all patients were immunosuppressed with tacrolimus and one of the anti-metabolites (mycophenolate mofetil (MMF) acid or azathioprine). In addition, one patient had atopic dermatitis, which itself causes predisposition to cutaneous warts by HPV. Only one patient was immunized with the HPV vaccine and the remaining 7 patients were unimmunized.

Cimetidine is postulated to act as an immunomodulatory agent at high doses by inhibiting suppressor T-cell function [15]. The paradigm between T helper (Th) 2 cells, and Th1 cells predominance is reflected in the level of cytokines that are released. Cimetidine activates Th1 cells to produce interleukin (IL)-2, IL-12, tumor necrosis factor (TNF)-α, and interferon (IFN)-γ and their expression correlates with improvement in cellular immunity and wart remission [16,17]. Patients who received cimetidine were shown to exhibit enhanced cell-mediated immunity, restoration of sensitivity following development of acquired tolerance, and increased response of lymphocytes to mitogen stimulation [18]. Although both cimetidine and ranitidine have been documented to have clinically significant immunomodulatory effects [19,20], ranitidine has shown only limited efficacy in a single open-label trial by Karaman et al [21]. 

In our study, cimetidine treatment for multiple recalcitrant cutaneous warts in pediatric heart transplant recipients showed promising results in all but one patient, who had additional condyloma accuminata, due to findings of HPV types 6 and 11. HPV type 1 commonly infects the soles of foot and produces plantar warts, while HPV 6 and 11 infect anogenital area and cause condyloma accuminata [22].

The common adverse events of cimetidine include headache, dizziness, diarrhea, constipation, drowsiness, fatigue, headache, urticarial rash, alopecia, gynecomastia, breast tenderness, arthralgia, and myalgia. Rare side effects seen in clinical trial programs include anaphylaxis, hepatitis, interstitial nephritis, pancreatitis, and hypersensitive vasculitis [23]. In our study, one patient developed mild gynecomastia. Cimetidine is known to cause gynecomastia and breast tenderness due to its antiandrogen effect [24]. Only one patient had pruritus, most likely due to associated atopic dermatitis. Cimetidine can increase plasma levels of tacrolimus and cyclosporine by inhibiting certain cytochrome P450 enzymes as well as by competition of renal tubular secretion [23]. In our cohort, there was no significant change in tacrolimus trough level. Increase in serum transaminases and creatinine have been reported usually during the first week of treatment and usually non-progressive, returning to pre-treatment values during therapy or one week following cessation of medication [23]. In our cohort of 8 patients, there was no significant change in serum creatinine or transaminases. 

The optimal duration of cimetidine therapy is unknown. In our study, most patients had a positive response following a minimum of 5 months of therapy, whereas the patient who completed only 3 months relapsed immediately after stopping the medications. Our study infers that it is safe to use high dose cimetidine (30–40 mg/kg/day) in 2 divided doses for 5–6 months.

### Study Limitations

There are a number of limitations including the retrospective nature of data analysis. There is lack of standardization with regard to the dose of cimetidine administration, duration, and interval of treatment. Spontaneous resolution is a potential confounding factor, and hence it is a major limitation to establish the efficacy of cimetidine. The incidence of acute rejection or change in cardiac hemodynamics could be due to multiple other factors, which are beyond the scope of this study. Serum drug levels were monitored according to patient visits but not obtained as pre-scheduled before and after cimetidine therapy in all patients. Similarly, the data for renal and liver function results are approximately obtained before and after cimetidine therapy. However, this is a single institution study involving heart transplant recipients who received a uniform immunosuppression therapy and followed with a rigorous clinical protocol driven management.

## 5. Conclusions

Since many conventional treatments for warts are painful, expensive, and may cause scarring, cimetidine offers a safe alternative treatment for cutaneous warts in pediatric heart transplant recipients.

## Figures and Tables

**Figure 1:**
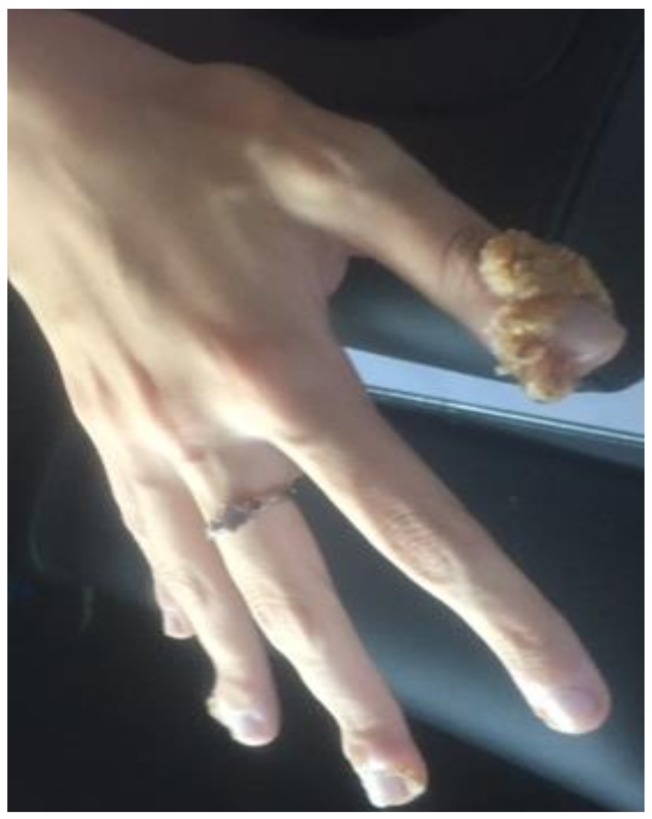
Verruca vulgaris in hand (Patient #1).

**Figure 2:**
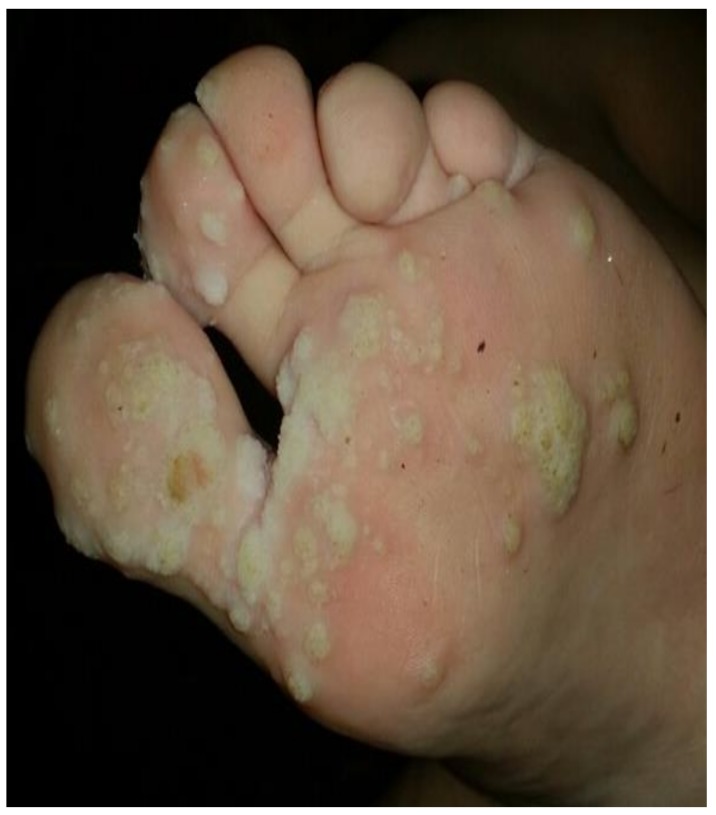
Verruca plantaris (Patient #2).

**Figure 3:**
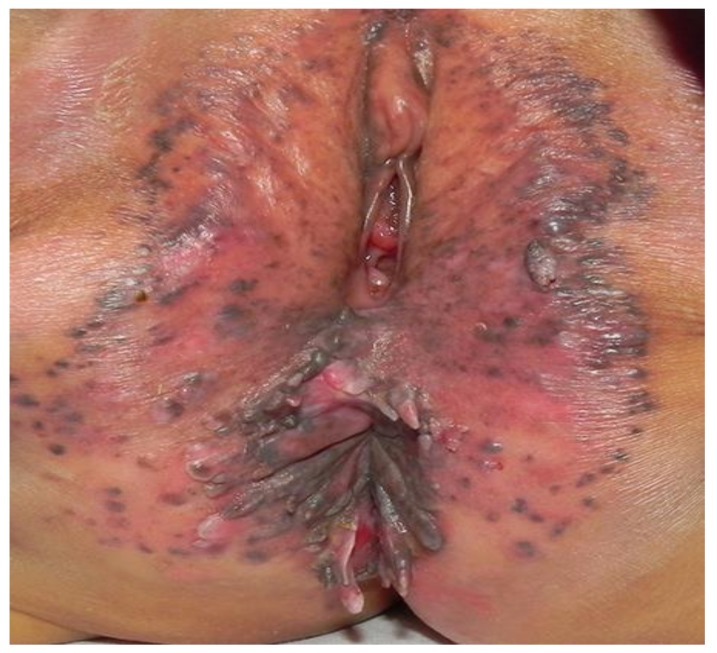
Anogenital warts (condyloma acuminata) (Patient #7).

**Table 1:** Clinical Summary.

Patient	Age in years.	Sex	Yrs. after HT	Immunosuppression Rx	Types of Warts	Rx with Cimeti-Dine in Months	Adverse Effects	Outcome of Warts after 6 Months	Outcome of Warts at 1 year. Follow-up
1	18	F	1	T + MMF	Verruca Vulgaris (Figure 1)	5	None	Resolved	No-recurrence
2	9	F	2	T + MMF	Verruca plantaris (Figure 2)	6	None	Resolved	Recurrence of warts, resolved after 3 months re-treatment
3	15	F	3	T + MMF	Verruca vulgaris	5	Pruritus	Resolved	No-recurrence
4	16	M	6	T + MMF	Verruca vulgaris	6	Gynecomastia	Resolved	No-recurrence
5	13	F	3	T + MMF	Verruca vulgaris	6	None	Resolved	No-recurrence
6	7	F	4	T + MMF	Verruca vulgaris	6	None	Resolved	No-recurrence
7	7	F	5	T + AZ	Verruca Vulgaris & Condyloma accuminata (Figure 3)	6	None	No effect	No effect
8	10	M	9	T + MMF	Verruca vulgaris	3	None	Recurred at end of 3 months Rx	Resolved after 6 months re-treatment with cimetidine

HT = heart transplant; T = tacrolimus; MMF = mycophenolic acid, AZ = azathioprine; Rx = therapy; F = female; M = male.
